# Supplementary material for: Modeling glioblastoma heterogeneity as a dynamic network of cell states
Source: Mol Syst Biol. 2021 Sep 16;17(9):e10105. doi: 10.15252/msb.202010105 (PMC8444284; doi:10.15252/msb.202010105)
Supplement: Supplementary file 6 — Source Data for Figure 5 [file MSB-17-e10105-s004.zip › Figure5A_sourcedata/GSEA_3017/hallmarks_stateE.GseaPreranked.1621934677158/HALLMARK_EPITHELIAL_MESENCHYMAL_TRANSITION.html]

Details for gene set HALLMARK\_EPITHELIAL\_MESENCHYMAL\_TRANSITION[GSEA]

|  || Dataset | state63017 |
| Phenotype | NoPhenotypeAvailable |
| Upregulated in class | na\_pos |
| GeneSet | HALLMARK\_EPITHELIAL\_MESENCHYMAL\_TRANSITION |
| Enrichment Score (ES) | 0.6411268 |
| Normalized Enrichment Score (NES) | 3.778121 |
| Nominal p-value | 0.0 |
| FDR q-value | 0.0 |
| FWER p-Value | 0.0 |
Table: GSEA Results Summary

  

Fig 1: Enrichment plot: HALLMARK\_EPITHELIAL\_MESENCHYMAL\_TRANSITION      
 Profile of the Running ES Score & Positions of GeneSet Members on the Rank Ordered List

  

| PROBE | GENE SYMBOL | GENE\_TITLE | RANK IN GENE LIST | RANK METRIC SCORE | RUNNING ES | CORE ENRICHMENT || 1 | PMP22 |  |  | 0 | 1.393 | 0.0879 | Yes |
| 2 | CD44 |  |  | 14 | 0.806 | 0.1245 | Yes |
| 3 | CDH2 |  |  | 27 | 0.668 | 0.1535 | Yes |
| 4 | GJA1 |  |  | 33 | 0.618 | 0.1870 | Yes |
| 5 | BDNF |  |  | 34 | 0.610 | 0.2255 | Yes |
| 6 | CALD1 |  |  | 41 | 0.580 | 0.2555 | Yes |
| 7 | PMEPA1 |  |  | 44 | 0.544 | 0.2876 | Yes |
| 8 | GPC1 |  |  | 50 | 0.530 | 0.3155 | Yes |
| 9 | SERPINE1 |  |  | 52 | 0.526 | 0.3476 | Yes |
| 10 | MMP2 |  |  | 57 | 0.503 | 0.3749 | Yes |
| 11 | PTX3 |  |  | 60 | 0.496 | 0.4040 | Yes |
| 12 | CDH6 |  |  | 70 | 0.453 | 0.4227 | Yes |
| 13 | COL5A2 |  |  | 83 | 0.434 | 0.4369 | Yes |
| 14 | CD59 |  |  | 89 | 0.418 | 0.4578 | Yes |
| 15 | IGFBP2 |  |  | 102 | 0.395 | 0.4696 | Yes |
| 16 | TIMP3 |  |  | 106 | 0.391 | 0.4909 | Yes |
| 17 | TPM1 |  |  | 108 | 0.388 | 0.5143 | Yes |
| 18 | LGALS1 |  |  | 113 | 0.368 | 0.5331 | Yes |
| 19 | RHOB |  |  | 148 | 0.335 | 0.5170 | Yes |
| 20 | CADM1 |  |  | 159 | 0.326 | 0.5266 | Yes |
| 21 | DPYSL3 |  |  | 161 | 0.325 | 0.5460 | Yes |
| 22 | TFPI2 |  |  | 166 | 0.322 | 0.5620 | Yes |
| 23 | JUN |  |  | 172 | 0.316 | 0.5764 | Yes |
| 24 | FLNA |  |  | 197 | 0.298 | 0.5689 | Yes |
| 25 | SAT1 |  |  | 206 | 0.291 | 0.5785 | Yes |
| 26 | MEST |  |  | 215 | 0.285 | 0.5877 | Yes |
| 27 | FERMT2 |  |  | 219 | 0.283 | 0.6023 | Yes |
| 28 | SCG2 |  |  | 220 | 0.283 | 0.6201 | Yes |
| 29 | PRRX1 |  |  | 229 | 0.278 | 0.6289 | Yes |
| 30 | SPP1 |  |  | 261 | 0.257 | 0.6112 | Yes |
| 31 | TNFRSF12A |  |  | 262 | 0.257 | 0.6274 | Yes |
| 32 | PFN2 |  |  | 265 | 0.253 | 0.6411 | Yes |
| 33 | SERPINE2 |  |  | 585 | -0.351 | 0.3135 | No |
| 34 | MCM7 |  |  | 817 | -0.599 | 0.0980 | No |
| 35 | GAS1 |  |  | 839 | -0.671 | 0.1173 | No |
Table: GSEA details [plain text format]

  

Fig 2: HALLMARK\_EPITHELIAL\_MESENCHYMAL\_TRANSITION: Random ES distribution      
 Gene set null distribution of ES for **HALLMARK\_EPITHELIAL\_MESENCHYMAL\_TRANSITION**
